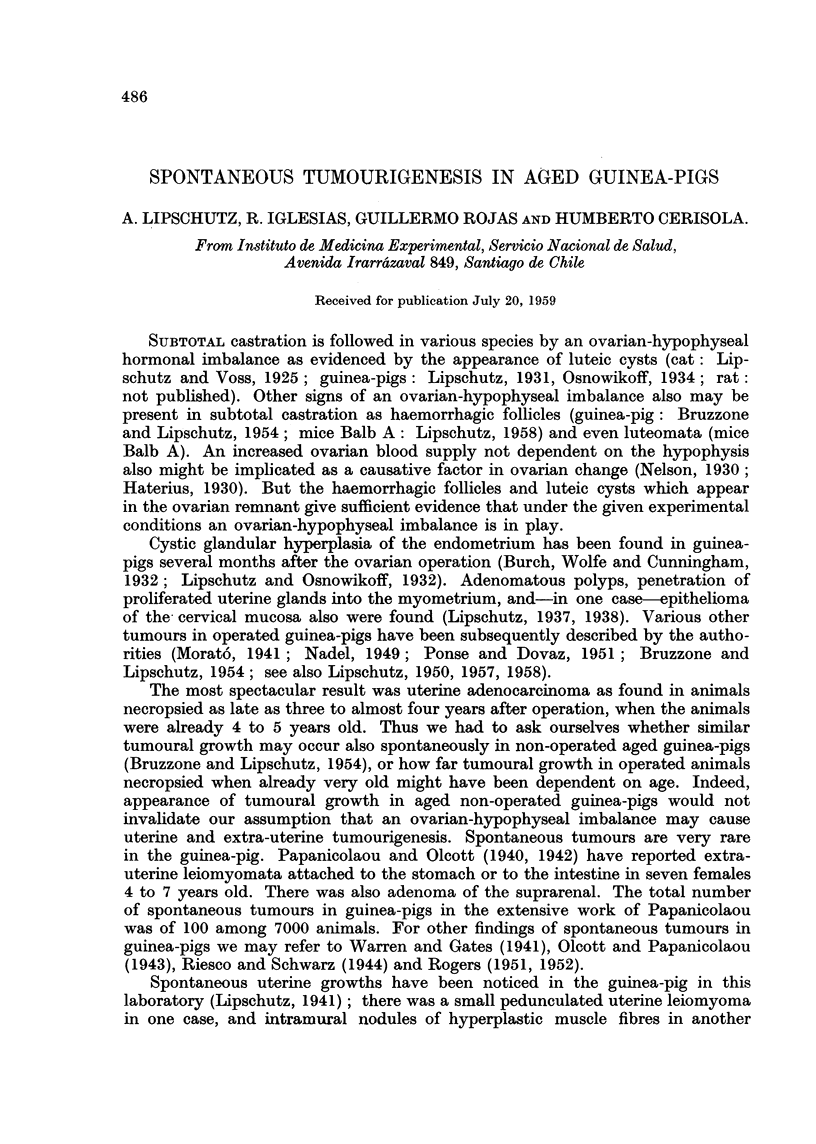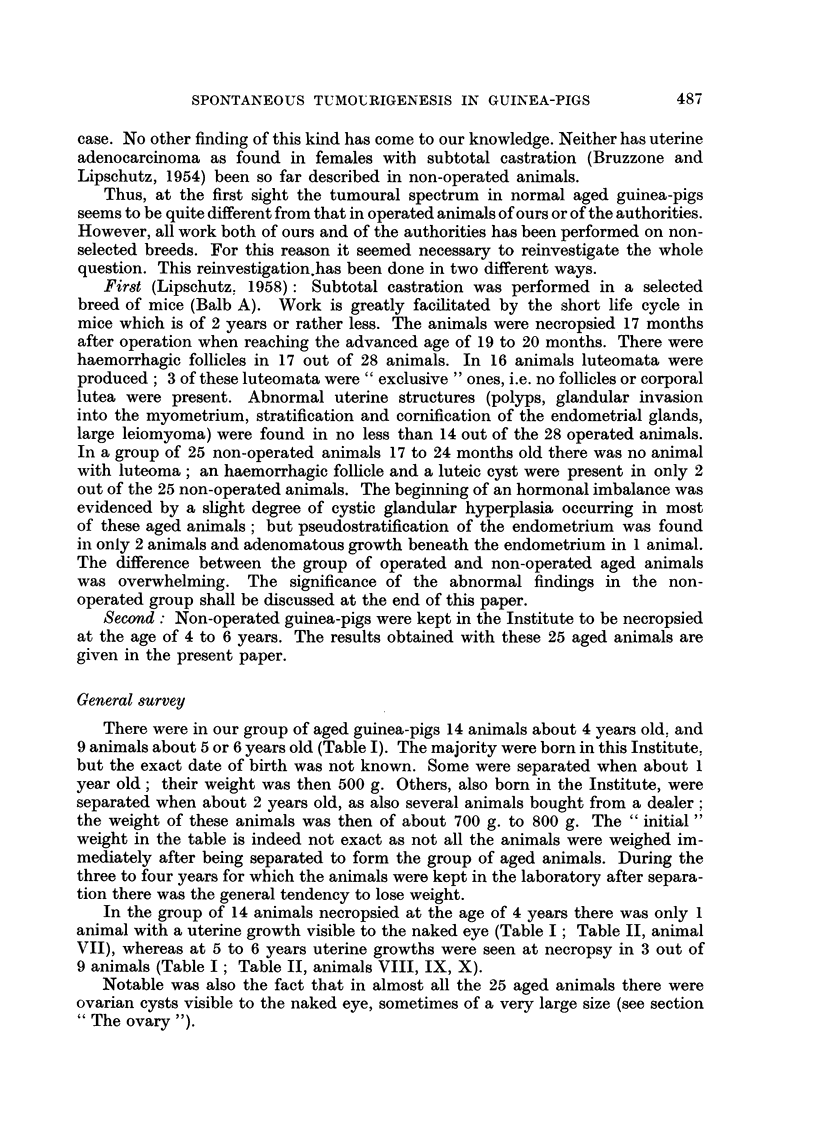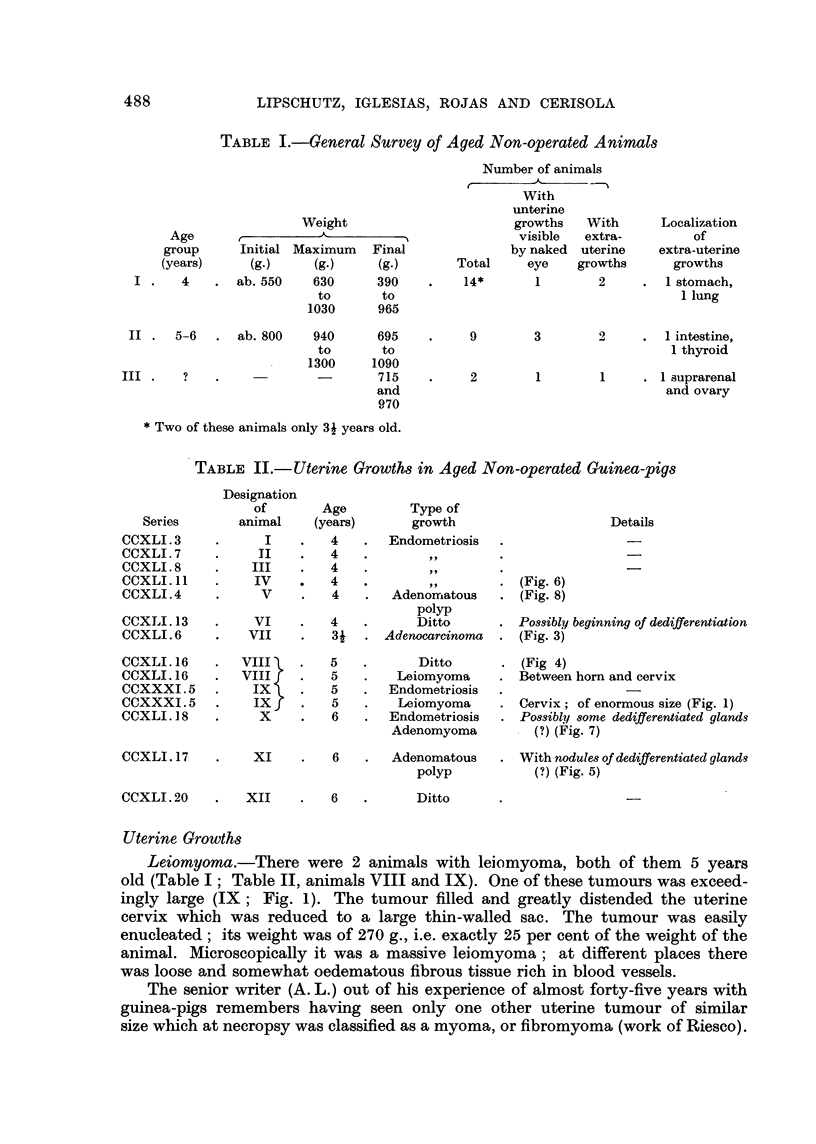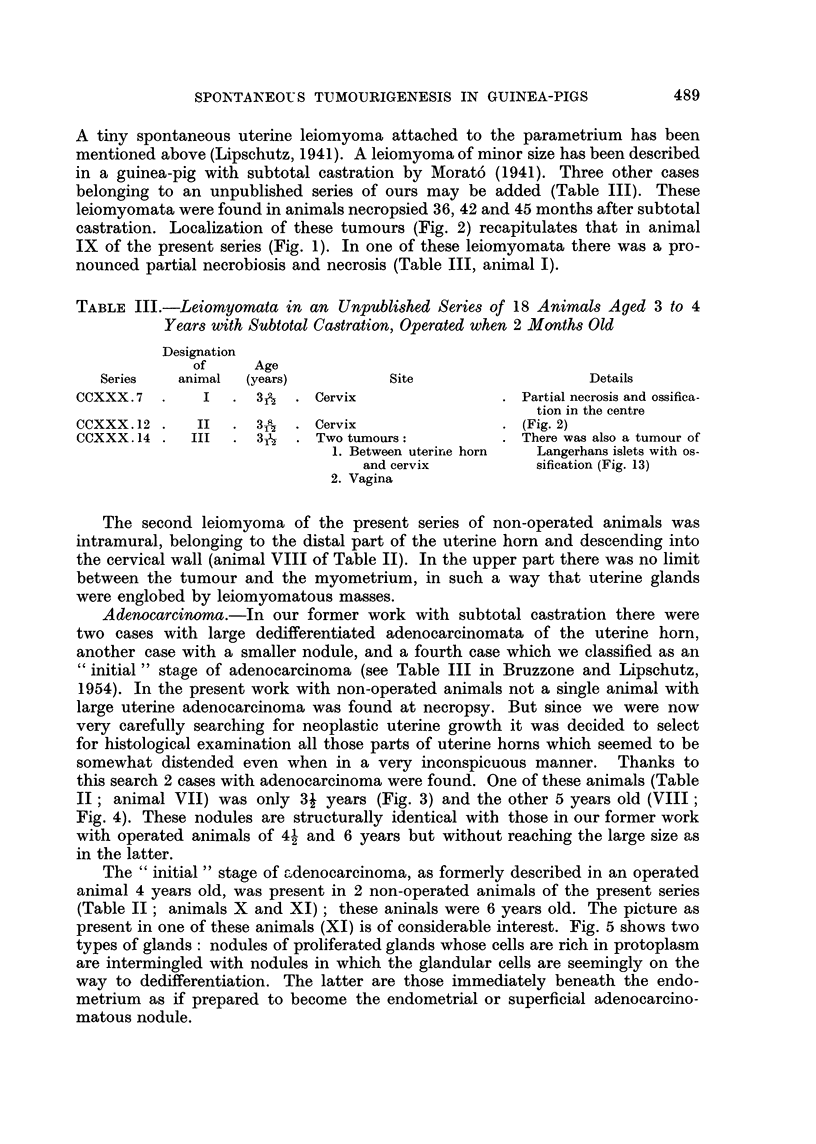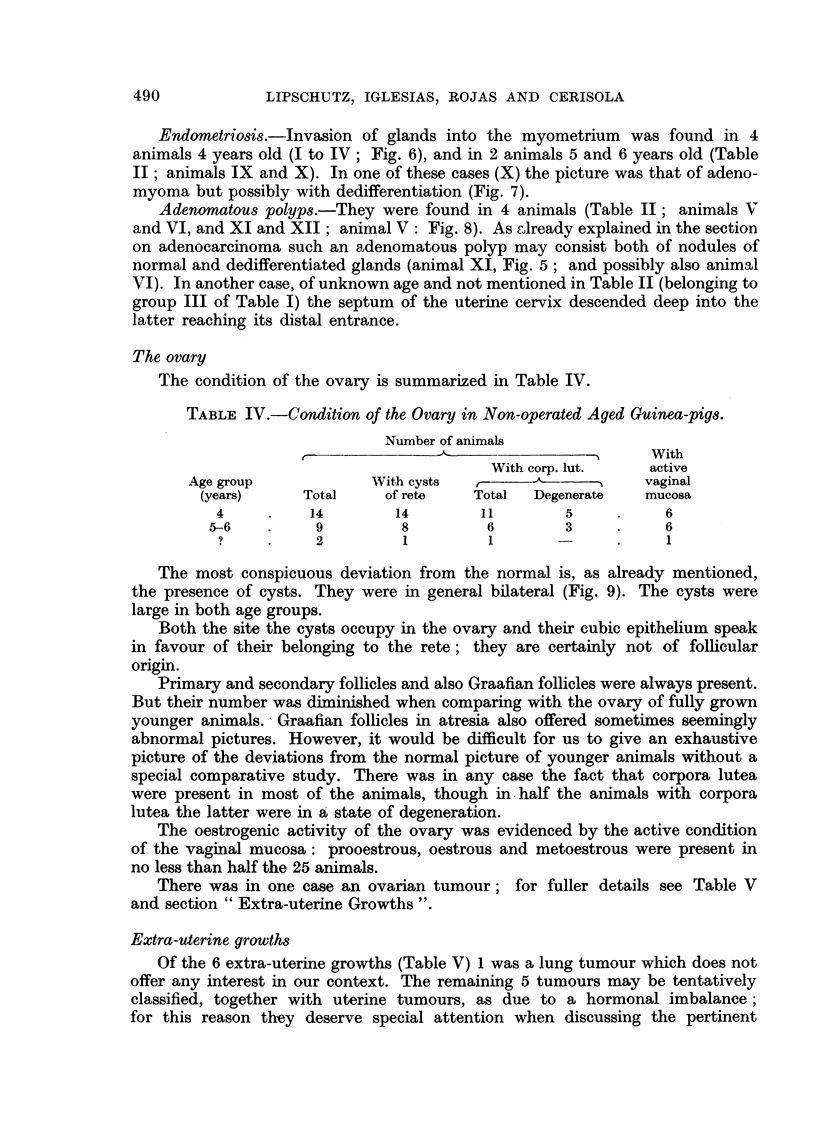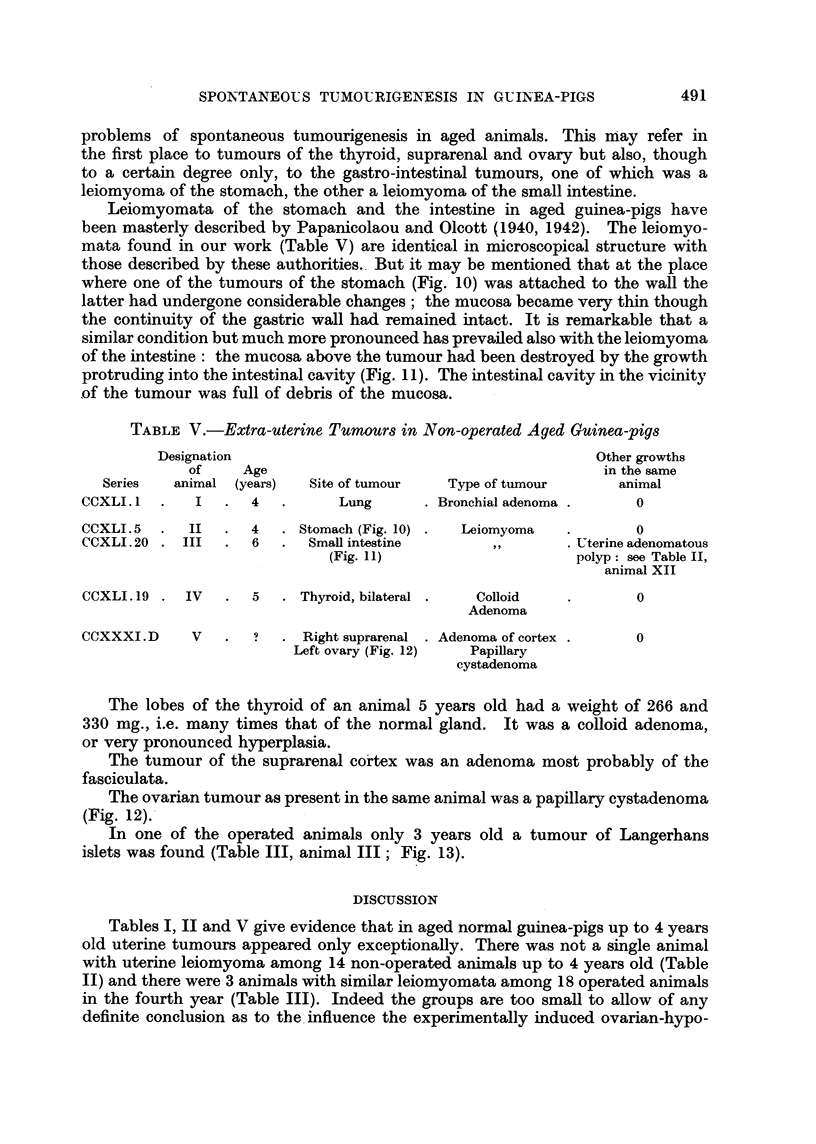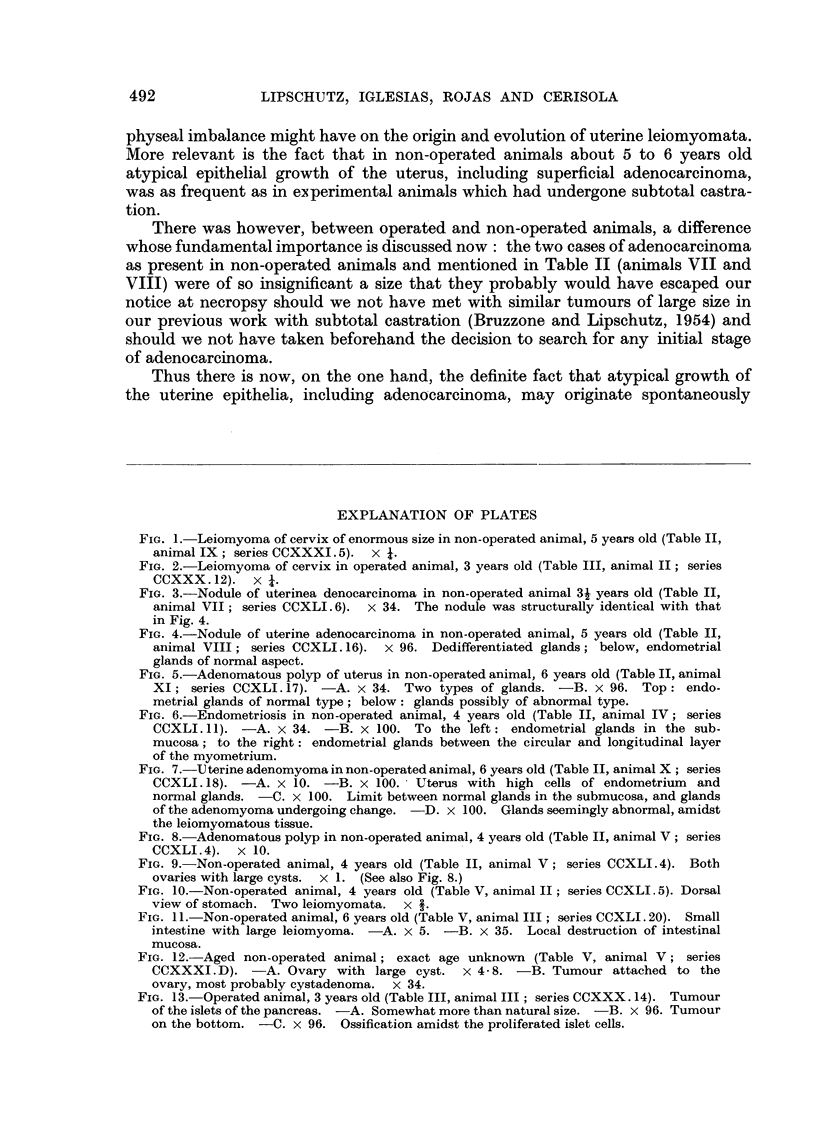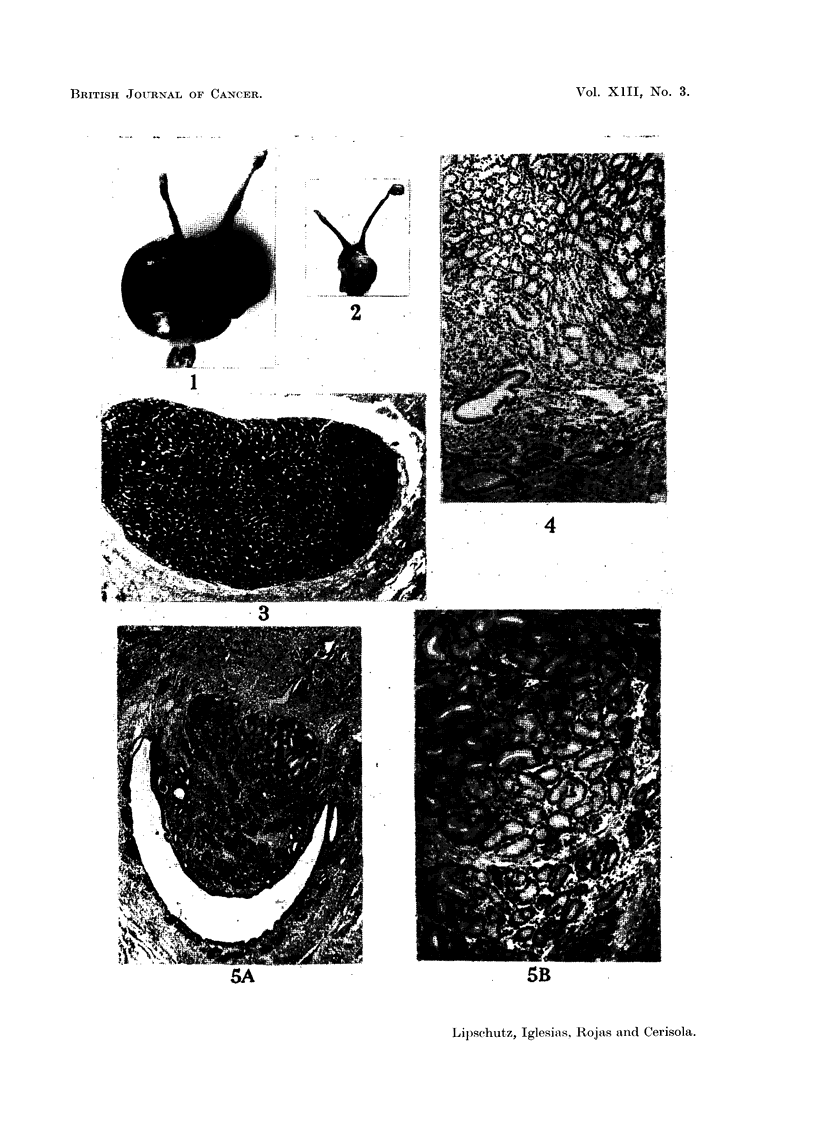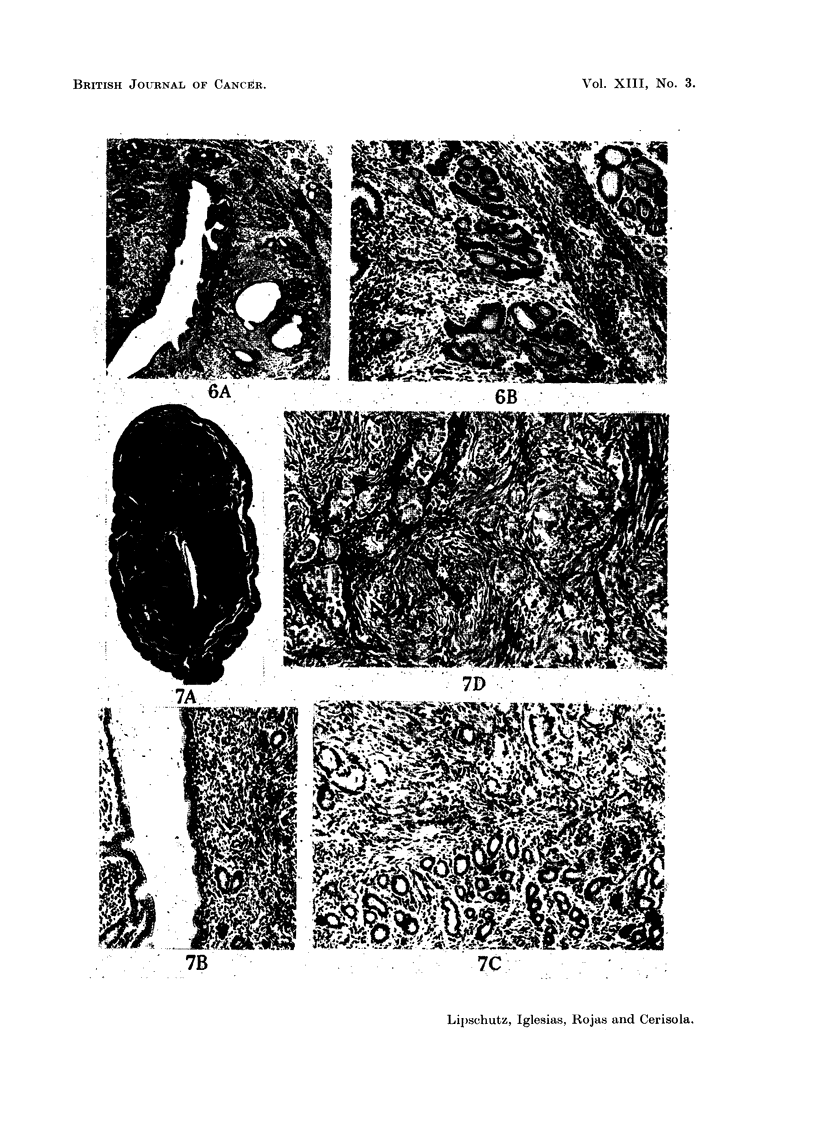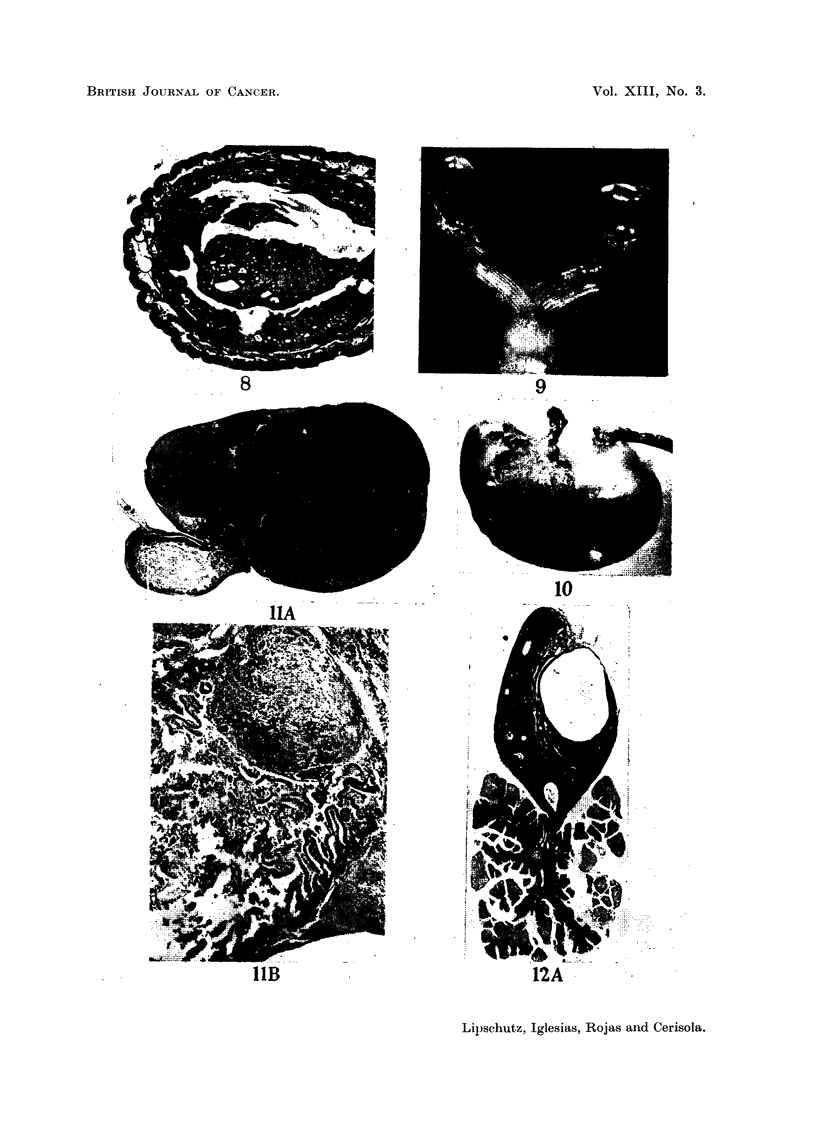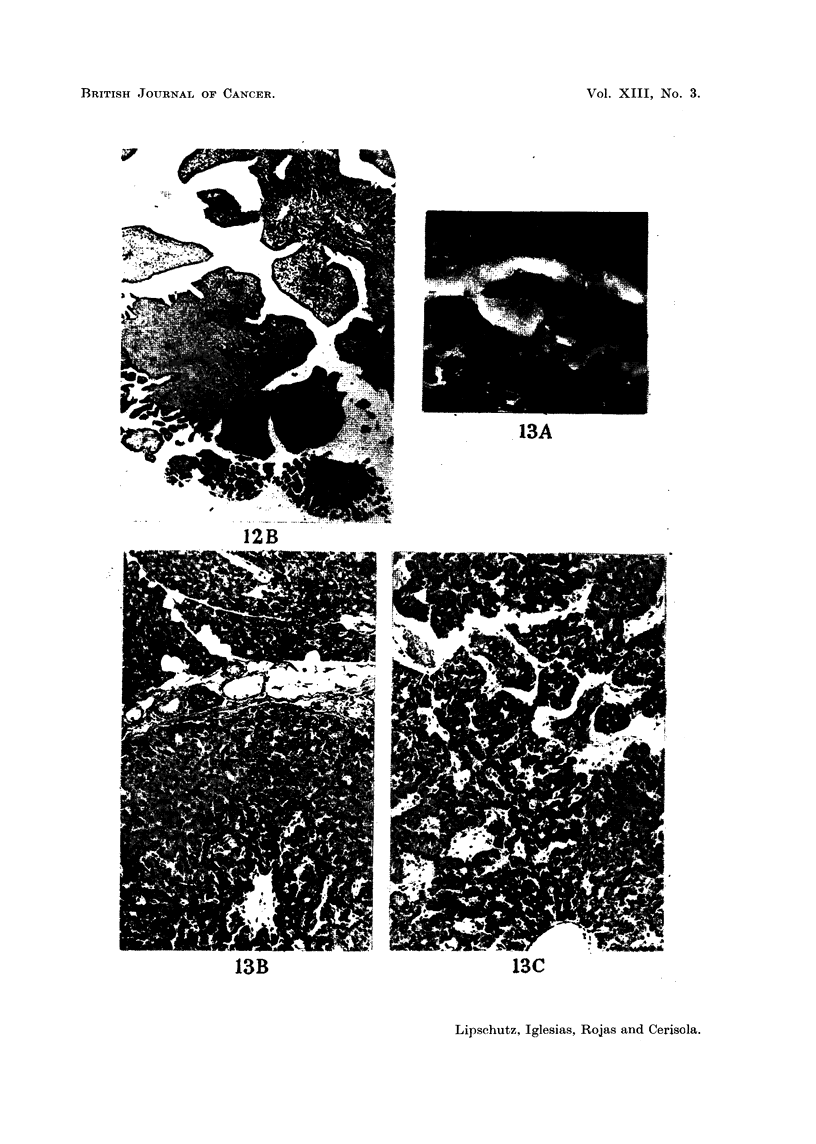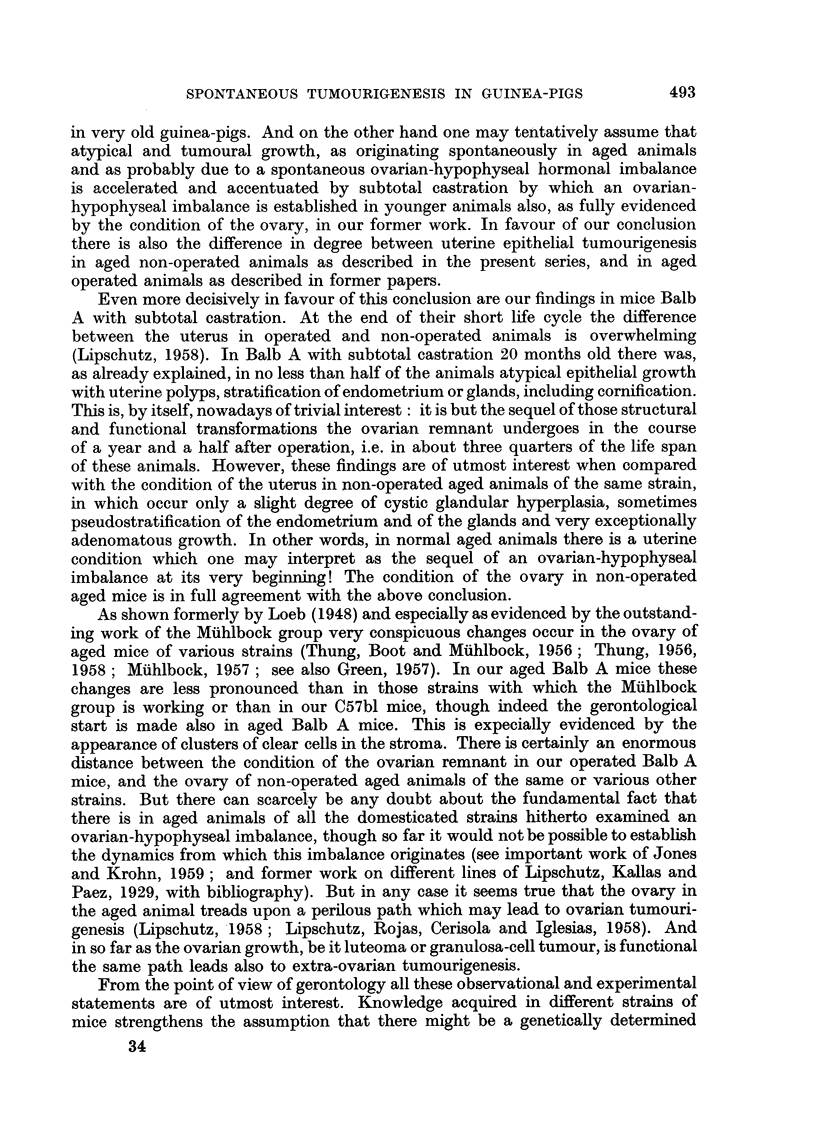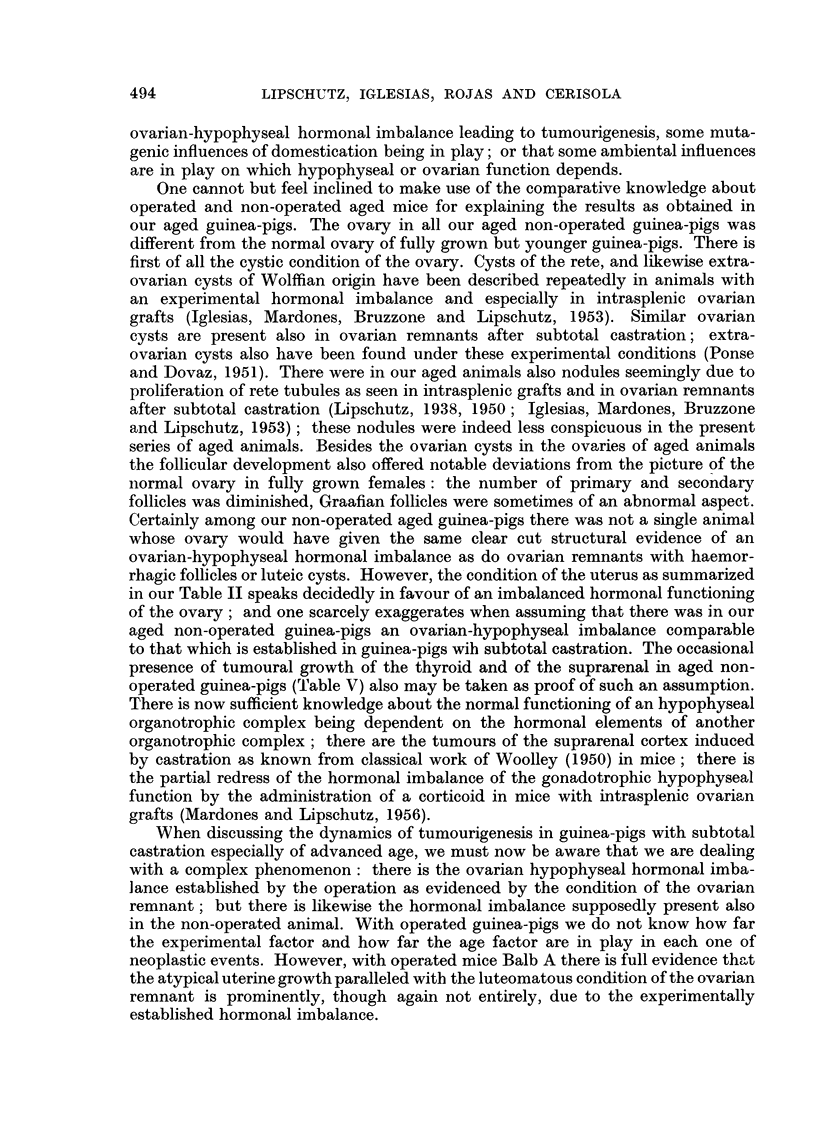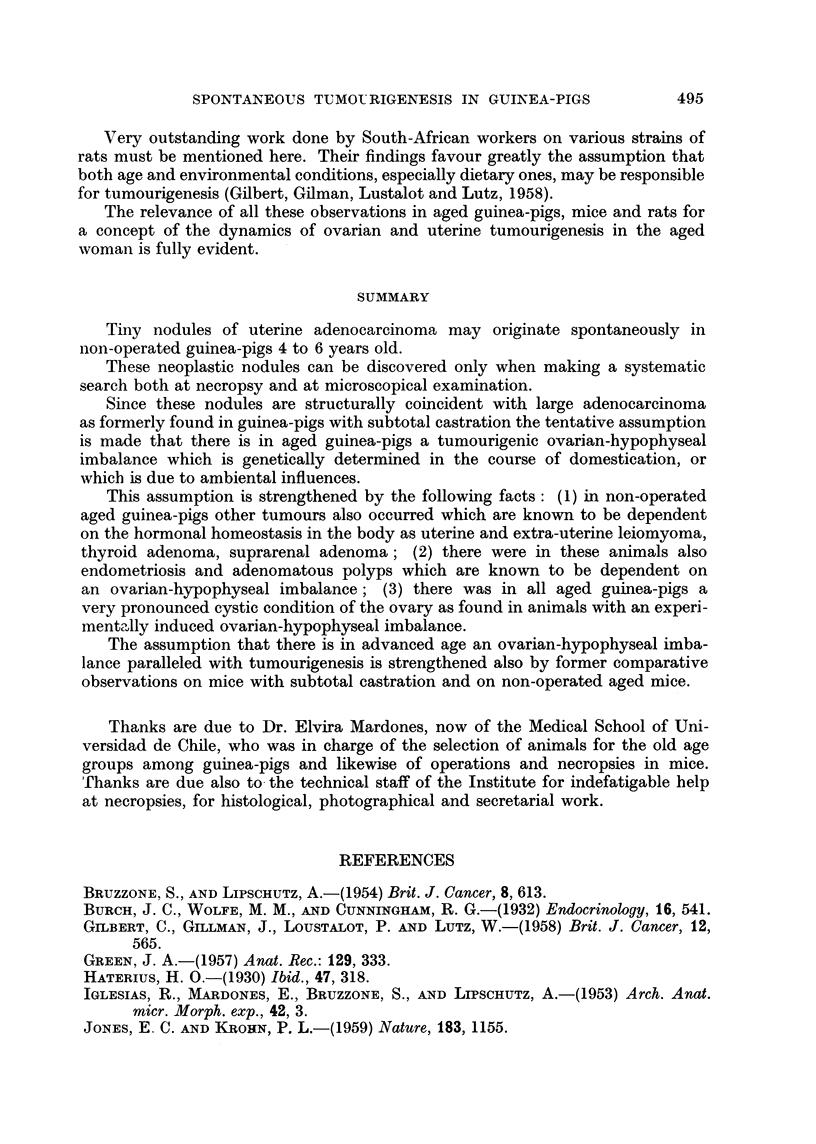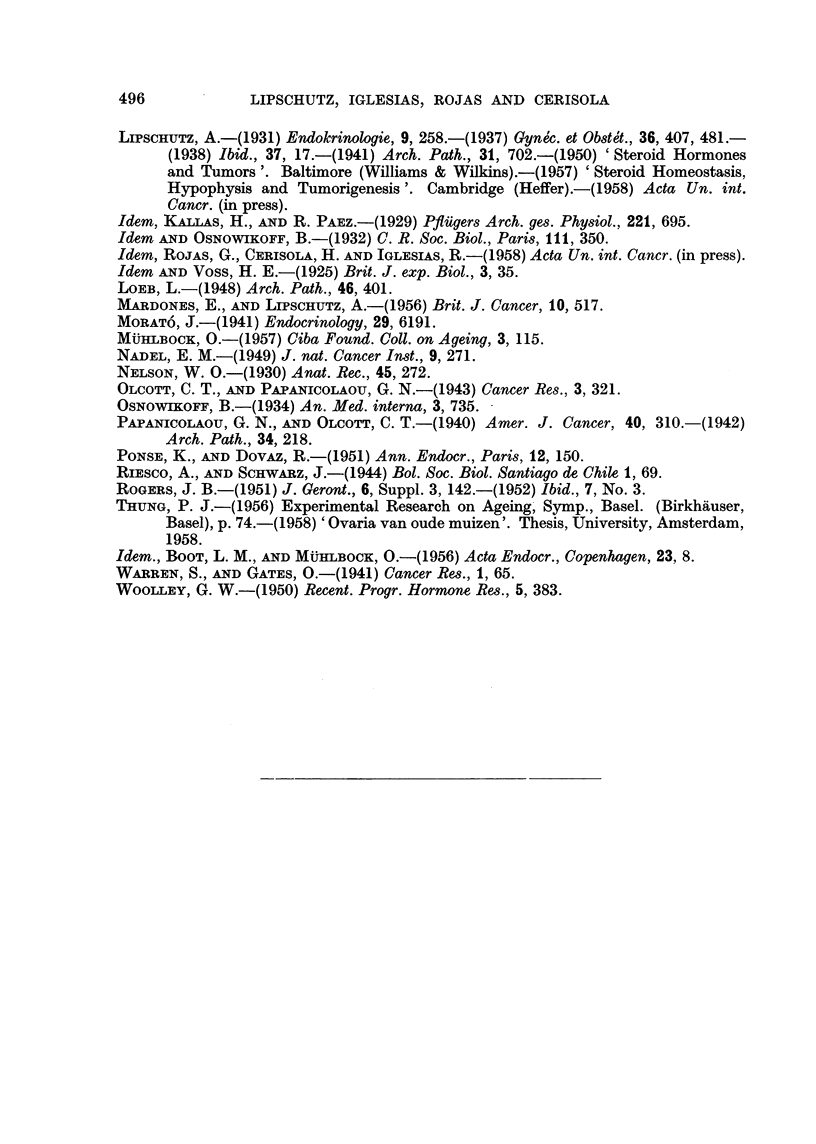# Spontaneous Tumourigenesis in Aged Guinea-pigs

**DOI:** 10.1038/bjc.1959.54

**Published:** 1959-09

**Authors:** A. Lipschutz, R. Iglesias, Guillermo Rojas, Humberto Cerisola

## Abstract

**Images:**


### SPONTANEOUS TUMOURIGENESIS IN AGED GUINEA-PIGS

A. LIPSCHUTZ, R. IGLESIAS, GUILLERMO ROJAS AND HUMBERTO CERISOLA.

*From Instituto de Medicina Experimental, Servicio Nacional de Salud, Avenida Irarrázaval* 849, *Santiago de Chile*

Received for publication July 20, 1959

SUBTOTAL castration is followed in various species by an ovarian-hypophyseal hormonal imbalance as evidenced by the appearance of luteic cysts (cat: Lipschutz and Voss, 1925; guinea-pigs: Lipschutz, 1931, Osnowikoff, 1934; rat: not published). Other signs of an ovarian-hypophyseal imbalance also may be present in subtotal castration as haemorrhagic follicles (guinea-pig: Bruzzone and Lipschutz, 1954; mice Balb A: Lipschutz, 1958) and even luteomata (mice Balb A). An increased ovarian blood supply not dependent on the hypophysis also might be implicated as a causative factor in ovarian change (Nelson, 1930; Haterius, 1930). But the haemorrhagic follicles and luteic cysts which appear in the ovarian remnant give sufficient evidence that under the given experimental conditions an ovarian-hypophyseal imbalance is in play.

Cystic glandular hyperplasia of the endometrium has been found in guinea-pigs several months after the ovarian operation (Burch, Wolfe and Cunningham, 1932; Lipschutz and Osnowikoff, 1932). Adenomatous polyps, penetration of proliferated uterine glands into the myometrium, and—in one case—epithelioma of the cervical mucosa also were found (Lipschutz, 1937, 1938). Various other tumours in operated guinea-pigs have been subsequently described by the authorities (Morató, 1941; Nadel, 1949; Ponse and Dovaz, 1951; Bruzzone and Lipschutz, 1954; see also Lipschutz, 1950, 1957, 1958).

The most spectacular result was uterine adenocarcinoma as found in animals necropsied as late as three to almost four years after operation, when the animals were already 4 to 5 years old. Thus we had to ask ourselves whether similar tumoural growth may occur also spontaneously in non-operated aged guinea-pigs (Bruzzone and Lipschutz, 1954), or how far tumoural growth in operated animals necropsied when already very old might have been dependent on age. Indeed, appearance of tumoural growth in aged non-operated guinea-pigs would not invalidate our assumption that an ovarian-hypophyseal imbalance may cause uterine and extra-uterine tumourigenesis. Spontaneous tumours are very rare in the guinea-pig. Papanicolaou and Olcott (1940, 1942) have reported extra-uterine leiomyomata attached to the stomach or to the intestine in seven females 4 to 7 years old. There was also adenoma of the suprarenal. The total number of spontaneous tumours in guinea-pigs in the extensive work of Papanicolaou was of 100 among 7000 animals. For other findings of spontaneous tumours in guinea-pigs we may refer to Warren and Gates (1941), Olcott and Papanicolaou (1943), Riesco and Schwarz (1944) and Rogers (1951, 1952).

Spontaneous uterine growths have been noticed in the guinea-pig in this laboratory (Lipschutz, 1941); there was a small pedunculated uterine leiomyoma in one case, and intramural nodules of hyperplastic muscle fibres in another case. No other finding of this kind has come to our knowledge. Neither has uterine adenocarcinoma as found in females with subtotal castration (Bruzzone and Lipschutz, 1954) been so far described in non-operated animals.

Thus, at the first sight the tumoural spectrum in normal aged guinea-pigs seems to be quite different from that in operated animals of ours or of the authorities. However, all work both of ours and of the authorities has been performed on non-selected breeds. For this reason it seemed necessary to reinvestigate the whole question. This reinvestigation has been done in two different ways.

*First* (Lipschutz, 1958): Subtotal castration was performed in a selected breed of mice (Balb A). Work is greatly facilitated by the short life cycle in mice which is of 2 years or rather less. The animals were necropsied 17 months after operation when reaching the advanced age of 19 to 20 months. There were haemorrhagic follicles in 17 out of 28 animals. In 16 animals luteomata were produced ; 3 of these luteomata were " exclusive " ones, i.e. no follicles or corporal lutea were present. Abnormal uterine structures (polyps, glandular invasion into the myometrium, stratification and cornification of the endometrial glands, large leiomyoma) were found in no less than 14 out of the 28 operated animals. In a group of 25 non-operated animals 17 to 24 months old there was no animal with luteoma ; an haemorrhagic follicle and a luteic cyst were present in only 2 out of the 25 non-operated animals. The beginning of an hormonal imbalance was evidenced by a slight degree of cystic glandular hyperplasia occurring in most of these aged animals ; but pseudostratification of the endometrium was found in only 2 animals and adenomatous growth beneath the endometrium in 1 animal. The difference between the group of operated and non-operated aged animals was overwhelming. The significance of the abnormal findings in the non-operated group shall be discussed at the end of this paper.

*Second :* Non-operated guinea-pigs were kept in the Institute to be necropsied at the age of 4 to 6 years. The results obtained with these 25 aged animals are given in the present paper.

*General survey*

There were in our group of aged guinea-pigs 14 animals about 4 years old, and 9 animals about 5 or 6 years old (Table I). The majority were born in this Institute, but the exact date of birth was not known. Some were separated when about 1 year old ; their weight was then 500 g. Others, also born in the Institute, were separated when about 2 years old, as also several animals bought from a dealer ; the weight of these animals was then of about 700 g. to 800 g. The " initial " weight in the table is indeed not exact as not all the animals were weighed immediately after being separated to form the group of aged animals. During the three to four years for which the animals were kept in the laboratory after separation there was the general tendency to lose weight.

In the group of 14 animals necropsied at the age of 4 years there was only 1 animal with a uterine growth visible to the naked eye (Table I ; Table II, animal VII), whereas at 5 to 6 years uterine growths were seen at necropsy in 3 out of 9 animals (Table I ; Table II, animals VIII, IX, X).

Notable was also the fact that in almost all the 25 aged animals there were ovarian cysts visible to the naked eye, sometimes of a very large size (see section " The ovary ").

TABLE I.—*General Survey of Aged Non-operated Animals*

| | Age group (years) | Weight: Initial (g.) | Weight: Maximum (g.) | Weight: Final (g.) | Number of animals: Total | Number of animals: With unterine growths visible by naked eye | Number of animals: With extra-uterine growths | Localization of extra-uterine growths |
|---|---|---|---|---|---|---|---|---|
| I | 4 | ab. 550 | 630 to 1030 | 390 to 965 | 14* | 1 | 2 | 1 stomach, 1 lung |
| II | 5–6 | ab. 800 | 940 to 1300 | 695 to 1090 | 9 | 3 | 2 | 1 intestine, 1 thyroid |
| III | ? | — | — | 715 and 970 | 2 | 1 | 1 | 1 suprarenal and ovary |

* Two of these animals only 3½ years old.

TABLE II.—*Uterine Growths in Aged Non-operated Guinea-pigs*

| Series | Designation of animal | Age (years) | Type of growth | Details |
|---|---|---|---|---|
| CCXLI.3 | I | 4 | Endometriosis | — |
| CCXLI.7 | II | 4 | ,, | — |
| CCXLI.8 | III | 4 | ,, | — |
| CCXLI.11 | IV | 4 | ,, | (Fig. 6) |
| CCXLI.4 | V | 4 | Adenomatous polyp | (Fig. 8) |
| CCXLI.13 | VI | 4 | Ditto | *Possibly beginning of dedifferentiation* |
| CCXLI.6 | VII | 3½ | *Adenocarcinoma* | (Fig. 3) |
| CCXLI.16 | VIII | 5 | Ditto | (Fig 4) |
| CCXLI.16 | VIII | 5 | Leiomyoma | Between horn and cervix |
| CCXXXI.5 | IX | 5 | Endometriosis | — |
| CCXXXI.5 | IX | 5 | Leiomyoma | Cervix ; of enormous size (Fig. 1) |
| CCXLI.18 | X | 6 | Endometriosis Adenomyoma | *Possibly some dedifferentiated glands* (?) (Fig. 7) |
| CCXLI.17 | XI | 6 | Adenomatous polyp | With *nodules of dedifferentiated glands* (?) (Fig. 5) |
| CCXLI.20 | XII | 6 | Ditto | — |

*Uterine Growths*

*Leiomyoma.*—There were 2 animals with leiomyoma, both of them 5 years old (Table I ; Table II, animals VIII and IX). One of these tumours was exceedingly large (IX ; Fig. 1). The tumour filled and greatly distended the uterine cervix which was reduced to a large thin-walled sac. The tumour was easily enucleated ; its weight was of 270 g., i.e. exactly 25 per cent of the weight of the animal. Microscopically it was a massive leiomyoma ; at different places there was loose and somewhat oedematous fibrous tissue rich in blood vessels.

The senior writer (A. L.) out of his experience of almost forty-five years with guinea-pigs remembers having seen only one other uterine tumour of similar size which at necropsy was classified as a myoma, or fibromyoma (work of Riesco).

A tiny spontaneous uterine leiomyoma attached to the parametrium has been mentioned above (Lipschutz, 1941). A leiomyoma of minor size has been described in a guinea-pig with subtotal castration by Morató (1941). Three other cases belonging to an unpublished series of ours may be added (Table III). These leiomyomata were found in animals necropsied 36, 42 and 45 months after subtotal castration. Localization of these tumours (Fig. 2) recapitulates that in animal IX of the present series (Fig. 1). In one of these leiomyomata there was a pronounced partial necrobiosis and necrosis (Table III, animal I).

TABLE III.—*Leiomyomata in an Unpublished Series of* 18 *Animals Aged* 3 *to* 4 *Years with Subtotal Castration, Operated when* 2 *Months Old*

| Series | Designation of animal | Age (years) | Site | Details |
|---|---|---|---|---|
| CCXXX.7 | I | $3\frac{2}{12}$ | Cervix | Partial necrosis and ossification in the centre |
| CCXXX.12 | II | $3\frac{8}{12}$ | Cervix | (Fig. 2) |
| CCXXX.14 | III | $3\frac{1}{12}$ | Two tumours : 1. Between uterine horn and cervix 2. Vagina | There was also a tumour of Langerhans islets with ossification (Fig. 13) |

The second leiomyoma of the present series of non-operated animals was intramural, belonging to the distal part of the uterine horn and descending into the cervical wall (animal VIII of Table II). In the upper part there was no limit between the tumour and the myometrium, in such a way that uterine glands were englobed by leiomyomatous masses.

*Adenocarcinoma.*—In our former work with subtotal castration there were two cases with large dedifferentiated adenocarcinomata of the uterine horn, another case with a smaller nodule, and a fourth case which we classified as an " initial " stage of adenocarcinoma (see Table III in Bruzzone and Lipschutz, 1954). In the present work with non-operated animals not a single animal with large uterine adenocarcinoma was found at necropsy. But since we were now very carefully searching for neoplastic uterine growth it was decided to select for histological examination all those parts of uterine horns which seemed to be somewhat distended even when in a very inconspicuous manner. Thanks to this search 2 cases with adenocarcinoma were found. One of these animals (Table II ; animal VII) was only $3\frac{1}{2}$ years (Fig. 3) and the other 5 years old (VIII ; Fig. 4). These nodules are structurally identical with those in our former work with operated animals of $4\frac{1}{2}$ and 6 years but without reaching the large size as in the latter.

The " initial " stage of adenocarcinoma, as formerly described in an operated animal 4 years old, was present in 2 non-operated animals of the present series (Table II ; animals X and XI) ; these aninals were 6 years old. The picture as present in one of these animals (XI) is of considerable interest. Fig. 5 shows two types of glands : nodules of proliferated glands whose cells are rich in protoplasm are intermingled with nodules in which the glandular cells are seemingly on the way to dedifferentiation. The latter are those immediately beneath the endometrium as if prepared to become the endometrial or superficial adenocarcinomatous nodule.

*Endometriosis.*—Invasion of glands into the myometrium was found in 4 animals 4 years old (I to IV ; Fig. 6), and in 2 animals 5 and 6 years old (Table II ; animals IX and X). In one of these cases (X) the picture was that of adenomyoma but possibly with dedifferentiation (Fig. 7).

*Adenomatous polyps.*—They were found in 4 animals (Table II ; animals V and VI, and XI and XII ; animal V : Fig. 8). As already explained in the section on adenocarcinoma such an adenomatous polyp may consist both of nodules of normal and dedifferentiated glands (animal XI, Fig. 5 ; and possibly also animal VI). In another case, of unknown age and not mentioned in Table II (belonging to group III of Table I) the septum of the uterine cervix descended deep into the latter reaching its distal entrance.

### *The ovary*

The condition of the ovary is summarized in Table IV.

TABLE IV.—*Condition of the Ovary in Non-operated Aged Guinea-pigs.*

| Age group (years) | Number of animals: Total | With cysts of rete | With corp. lut.: Total | With corp. lut.: Degenerate | With active vaginal mucosa |
|---|---|---|---|---|---|
| 4 | 14 | 14 | 11 | 5 | 6 |
| 5–6 | 9 | 8 | 6 | 3 | 6 |
| ? | 2 | 1 | 1 | — | 1 |

The most conspicuous deviation from the normal is, as already mentioned, the presence of cysts. They were in general bilateral (Fig. 9). The cysts were large in both age groups.

Both the site the cysts occupy in the ovary and their cubic epithelium speak in favour of their belonging to the rete ; they are certainly not of follicular origin.

Primary and secondary follicles and also Graafian follicles were always present. But their number was diminished when comparing with the ovary of fully grown younger animals. Graafian follicles in atresia also offered sometimes seemingly abnormal pictures. However, it would be difficult for us to give an exhaustive picture of the deviations from the normal picture of younger animals without a special comparative study. There was in any case the fact that corpora lutea were present in most of the animals, though in half the animals with corpora lutea the latter were in a state of degeneration.

The oestrogenic activity of the ovary was evidenced by the active condition of the vaginal mucosa : prooestrous, oestrous and metoestrous were present in no less than half the 25 animals.

There was in one case an ovarian tumour ; for fuller details see Table V and section " Extra-uterine Growths ".

### *Extra-uterine growths*

Of the 6 extra-uterine growths (Table V) 1 was a lung tumour which does not offer any interest in our context. The remaining 5 tumours may be tentatively classified, together with uterine tumours, as due to a hormonal imbalance ; for this reason they deserve special attention when discussing the pertinent problems of spontaneous tumourigenesis in aged animals. This may refer in the first place to tumours of the thyroid, suprarenal and ovary but also, though to a certain degree only, to the gastro-intestinal tumours, one of which was a leiomyoma of the stomach, the other a leiomyoma of the small intestine.

Leiomyomata of the stomach and the intestine in aged guinea-pigs have been masterly described by Papanicolaou and Olcott (1940, 1942). The leiomyomata found in our work (Table V) are identical in microscopical structure with those described by these authorities. But it may be mentioned that at the place where one of the tumours of the stomach (Fig. 10) was attached to the wall the latter had undergone considerable changes ; the mucosa became very thin though the continuity of the gastric wall had remained intact. It is remarkable that a similar condition but much more pronounced has prevailed also with the leiomyoma of the intestine : the mucosa above the tumour had been destroyed by the growth protruding into the intestinal cavity (Fig. 11). The intestinal cavity in the vicinity of the tumour was full of debris of the mucosa.

TABLE V.—*Extra-uterine Tumours in Non-operated Aged Guinea-pigs*

| Series | Designation of animal | Age (years) | Site of tumour | Type of tumour | Other growths in the same animal |
|---|---|---|---|---|---|
| CCXLI.1 | I | 4 | Lung | Bronchial adenoma | 0 |
| CCXLI.5 | II | 4 | Stomach (Fig. 10) | Leiomyoma | 0 |
| CCXLI.20 | III | 6 | Small intestine (Fig. 11) | ,, | Uterine adenomatous polyp : see Table II, animal XII |
| CCXLI.19 | IV | 5 | Thyroid, bilateral | Colloid Adenoma | 0 |
| CCXXXI.D | V | ? | Right suprarenal<br>Left ovary (Fig. 12) | Adenoma of cortex<br>Papillary cystadenoma | 0 |

The lobes of the thyroid of an animal 5 years old had a weight of 266 and 330 mg., i.e. many times that of the normal gland. It was a colloid adenoma, or very pronounced hyperplasia.

The tumour of the suprarenal cortex was an adenoma most probably of the fasciculata.

The ovarian tumour as present in the same animal was a papillary cystadenoma (Fig. 12).

In one of the operated animals only 3 years old a tumour of Langerhans islets was found (Table III, animal III ; Fig. 13).

### DISCUSSION

Tables I, II and V give evidence that in aged normal guinea-pigs up to 4 years old uterine tumours appeared only exceptionally. There was not a single animal with uterine leiomyoma among 14 non-operated animals up to 4 years old (Table II) and there were 3 animals with similar leiomyomata among 18 operated animals in the fourth year (Table III). Indeed the groups are too small to allow of any definite conclusion as to the influence the experimentally induced ovarian-hypophyseal imbalance might have on the origin and evolution of uterine leiomyomata. More relevant is the fact that in non-operated animals about 5 to 6 years old atypical epithelial growth of the uterus, including superficial adenocarcinoma, was as frequent as in experimental animals which had undergone subtotal castration.

There was however, between operated and non-operated animals, a difference whose fundamental importance is discussed now : the two cases of adenocarcinoma as present in non-operated animals and mentioned in Table II (animals VII and VIII) were of so insignificant a size that they probably would have escaped our notice at necropsy should we not have met with similar tumours of large size in our previous work with subtotal castration (Bruzzone and Lipschutz, 1954) and should we not have taken beforehand the decision to search for any initial stage of adenocarcinoma.

Thus there is now, on the one hand, the definite fact that atypical growth of the uterine epithelia, including adenocarcinoma, may originate spontaneously

---

### EXPLANATION OF PLATES

FIG. 1.—Leiomyoma of cervix of enormous size in non-operated animal, 5 years old (Table II, animal IX ; series CCXXXI.5). × ¼.

FIG. 2.—Leiomyoma of cervix in operated animal, 3 years old (Table III, animal II ; series CCXXX.12). × ½.

FIG. 3.—Nodule of uterinea denocarcinoma in non-operated animal 3½ years old (Table II, animal VII ; series CCXLI.6). × 34. The nodule was structurally identical with that in Fig. 4.

FIG. 4.—Nodule of uterine adenocarcinoma in non-operated animal, 5 years old (Table II, animal VIII ; series CCXLI.16). × 96. Dedifferentiated glands ; below, endometrial glands of normal aspect.

FIG. 5.—Adenomatous polyp of uterus in non-operated animal, 6 years old (Table II, animal XI ; series CCXLI.17). —A. × 34. Two types of glands. —B. × 96. Top : endometrial glands of normal type ; below : glands possibly of abnormal type.

FIG. 6.—Endometriosis in non-operated animal, 4 years old (Table II, animal IV ; series CCXLI.11). —A. × 34. —B. × 100. To the left : endometrial glands in the submucosa ; to the right : endometrial glands between the circular and longitudinal layer of the myometrium.

FIG. 7.—Uterine adenomyoma in non-operated animal, 6 years old (Table II, animal X ; series CCXLI.18). —A. × 10. —B. × 100. Uterus with high cells of endometrium and normal glands. —C. × 100. Limit between normal glands in the submucosa, and glands of the adenomyoma undergoing change. —D. × 100. Glands seemingly abnormal, amidst the leiomyomatous tissue.

FIG. 8.—Adenomatous polyp in non-operated animal, 4 years old (Table II, animal V ; series CCXLI.4). × 10.

FIG. 9.—Non-operated animal, 4 years old (Table II, animal V ; series CCXLI.4). Both ovaries with large cysts. × 1. (See also Fig. 8.)

FIG. 10.—Non-operated animal, 4 years old (Table V, animal II ; series CCXLI.5). Dorsal view of stomach. Two leiomyomata. × ¾.

FIG. 11.—Non-operated animal, 6 years old (Table V, animal III ; series CCXLI.20). Small intestine with large leiomyoma. —A. × 5. —B. × 35. Local destruction of intestinal mucosa.

FIG. 12.—Aged non-operated animal ; exact age unknown (Table V, animal V ; series CCXXXI.D). —A. Ovary with large cyst. × 4·8. —B. Tumour attached to the ovary, most probably cystadenoma. × 34.

FIG. 13.—Operated animal, 3 years old (Table III, animal III ; series CCXXX.14). Tumour of the islets of the pancreas. —A. Somewhat more than natural size. —B. × 96. Tumour on the bottom. —C. × 96. Ossification amidst the proliferated islet cells.

1

2

4

3

5A

5B

Lipschutz, Iglesias, Rojas and Cerisola.

6A

6B

7A

7D

7B

7C

Lipschutz, Iglesias, Rojas and Cerisola.

8

9

11A

10

11B

12A

12B

13A

13B

13C

in very old guinea-pigs. And on the other hand one may tentatively assume that atypical and tumoural growth, as originating spontaneously in aged animals and as probably due to a spontaneous ovarian-hypophyseal hormonal imbalance is accelerated and accentuated by subtotal castration by which an ovarian-hypophyseal imbalance is established in younger animals also, as fully evidenced by the condition of the ovary, in our former work. In favour of our conclusion there is also the difference in degree between uterine epithelial tumourigenesis in aged non-operated animals as described in the present series, and in aged operated animals as described in former papers.

Even more decisively in favour of this conclusion are our findings in mice Balb A with subtotal castration. At the end of their short life cycle the difference between the uterus in operated and non-operated animals is overwhelming (Lipschutz, 1958). In Balb A with subtotal castration 20 months old there was, as already explained, in no less than half of the animals atypical epithelial growth with uterine polyps, stratification of endometrium or glands, including cornification. This is, by itself, nowadays of trivial interest : it is but the sequel of those structural and functional transformations the ovarian remnant undergoes in the course of a year and a half after operation, i.e. in about three quarters of the life span of these animals. However, these findings are of utmost interest when compared with the condition of the uterus in non-operated aged animals of the same strain, in which occur only a slight degree of cystic glandular hyperplasia, sometimes pseudostratification of the endometrium and of the glands and very exceptionally adenomatous growth. In other words, in normal aged animals there is a uterine condition which one may interpret as the sequel of an ovarian-hypophyseal imbalance at its very beginning! The condition of the ovary in non-operated aged mice is in full agreement with the above conclusion.

As shown formerly by Loeb (1948) and especially as evidenced by the outstanding work of the Mühlbock group very conspicuous changes occur in the ovary of aged mice of various strains (Thung, Boot and Mühlbock, 1956 ; Thung, 1956, 1958 ; Mühlbock, 1957 ; see also Green, 1957). In our aged Balb A mice these changes are less pronounced than in those strains with which the Mühlbock group is working or than in our C57bl mice, though indeed the gerontological start is made also in aged Balb A mice. This is expecially evidenced by the appearance of clusters of clear cells in the stroma. There is certainly an enormous distance between the condition of the ovarian remnant in our operated Balb A mice, and the ovary of non-operated aged animals of the same or various other strains. But there can scarcely be any doubt about the fundamental fact that there is in aged animals of all the domesticated strains hitherto examined an ovarian-hypophyseal imbalance, though so far it would not be possible to establish the dynamics from which this imbalance originates (see important work of Jones and Krohn, 1959 ; and former work on different lines of Lipschutz, Kallas and Paez, 1929, with bibliography). But in any case it seems true that the ovary in the aged animal treads upon a perilous path which may lead to ovarian tumourigenesis (Lipschutz, 1958 ; Lipschutz, Rojas, Cerisola and Iglesias, 1958). And in so far as the ovarian growth, be it luteoma or granulosa-cell tumour, is functional the same path leads also to extra-ovarian tumourigenesis.

From the point of view of gerontology all these observational and experimental statements are of utmost interest. Knowledge acquired in different strains of mice strengthens the assumption that there might be a genetically determined ovarian-hypophyseal hormonal imbalance leading to tumourigenesis, some mutagenic influences of domestication being in play; or that some ambiental influences are in play on which hypophyseal or ovarian function depends.

One cannot but feel inclined to make use of the comparative knowledge about operated and non-operated aged mice for explaining the results as obtained in our aged guinea-pigs. The ovary in all our aged non-operated guinea-pigs was different from the normal ovary of fully grown but younger guinea-pigs. There is first of all the cystic condition of the ovary. Cysts of the rete, and likewise extra-ovarian cysts of Wolffian origin have been described repeatedly in animals with an experimental hormonal imbalance and especially in intrasplenic ovarian grafts (Iglesias, Mardones, Bruzzone and Lipschutz, 1953). Similar ovarian cysts are present also in ovarian remnants after subtotal castration; extra-ovarian cysts also have been found under these experimental conditions (Ponse and Dovaz, 1951). There were in our aged animals also nodules seemingly due to proliferation of rete tubules as seen in intrasplenic grafts and in ovarian remnants after subtotal castration (Lipschutz, 1938, 1950; Iglesias, Mardones, Bruzzone and Lipschutz, 1953); these nodules were indeed less conspicuous in the present series of aged animals. Besides the ovarian cysts in the ovaries of aged animals the follicular development also offered notable deviations from the picture of the normal ovary in fully grown females: the number of primary and secondary follicles was diminished, Graafian follicles were sometimes of an abnormal aspect. Certainly among our non-operated aged guinea-pigs there was not a single animal whose ovary would have given the same clear cut structural evidence of an ovarian-hypophyseal hormonal imbalance as do ovarian remnants with haemorrhagic follicles or luteic cysts. However, the condition of the uterus as summarized in our Table II speaks decidedly in favour of an imbalanced hormonal functioning of the ovary; and one scarcely exaggerates when assuming that there was in our aged non-operated guinea-pigs an ovarian-hypophyseal imbalance comparable to that which is established in guinea-pigs wih subtotal castration. The occasional presence of tumoural growth of the thyroid and of the suprarenal in aged non-operated guinea-pigs (Table V) also may be taken as proof of such an assumption. There is now sufficient knowledge about the normal functioning of an hypophyseal organotrophic complex being dependent on the hormonal elements of another organotrophic complex; there are the tumours of the suprarenal cortex induced by castration as known from classical work of Woolley (1950) in mice; there is the partial redress of the hormonal imbalance of the gonadotrophic hypophyseal function by the administration of a corticoid in mice with intrasplenic ovarian grafts (Mardones and Lipschutz, 1956).

When discussing the dynamics of tumourigenesis in guinea-pigs with subtotal castration especially of advanced age, we must now be aware that we are dealing with a complex phenomenon: there is the ovarian hypophyseal hormonal imbalance established by the operation as evidenced by the condition of the ovarian remnant; but there is likewise the hormonal imbalance supposedly present also in the non-operated animal. With operated guinea-pigs we do not know how far the experimental factor and how far the age factor are in play in each one of neoplastic events. However, with operated mice Balb A there is full evidence that the atypical uterine growth paralleled with the luteomatous condition of the ovarian remnant is prominently, though again not entirely, due to the experimentally established hormonal imbalance.

Very outstanding work done by South-African workers on various strains of rats must be mentioned here. Their findings favour greatly the assumption that both age and environmental conditions, especially dietary ones, may be responsible for tumourigenesis (Gilbert, Gilman, Lustalot and Lutz, 1958).

The relevance of all these observations in aged guinea-pigs, mice and rats for a concept of the dynamics of ovarian and uterine tumourigenesis in the aged woman is fully evident.

### SUMMARY

Tiny nodules of uterine adenocarcinoma may originate spontaneously in non-operated guinea-pigs 4 to 6 years old.

These neoplastic nodules can be discovered only when making a systematic search both at necropsy and at microscopical examination.

Since these nodules are structurally coincident with large adenocarcinoma as formerly found in guinea-pigs with subtotal castration the tentative assumption is made that there is in aged guinea-pigs a tumourigenic ovarian-hypophyseal imbalance which is genetically determined in the course of domestication, or which is due to ambiental influences.

This assumption is strengthened by the following facts : (1) in non-operated aged guinea-pigs other tumours also occurred which are known to be dependent on the hormonal homeostasis in the body as uterine and extra-uterine leiomyoma, thyroid adenoma, suprarenal adenoma ; (2) there were in these animals also endometriosis and adenomatous polyps which are known to be dependent on an ovarian-hypophyseal imbalance ; (3) there was in all aged guinea-pigs a very pronounced cystic condition of the ovary as found in animals with an experimentally induced ovarian-hypophyseal imbalance.

The assumption that there is in advanced age an ovarian-hypophyseal imbalance paralleled with tumourigenesis is strengthened also by former comparative observations on mice with subtotal castration and on non-operated aged mice.

Thanks are due to Dr. Elvira Mardones, now of the Medical School of Universidad de Chile, who was in charge of the selection of animals for the old age groups among guinea-pigs and likewise of operations and necropsies in mice. Thanks are due also to the technical staff of the Institute for indefatigable help at necropsies, for histological, photographical and secretarial work.

### REFERENCES

BRUZZONE, S., AND LIPSCHUTZ, A.—(1954) *Brit. J. Cancer*, **8**, 613.
BURCH, J. C., WOLFE, M. M., AND CUNNINGHAM, R. G.—(1932) *Endocrinology*, **16**, 541.
GILBERT, C., GILLMAN, J., LOUSTALOT, P. AND LUTZ, W.—(1958) *Brit. J. Cancer*, **12**, 565.
GREEN, J. A.—(1957) *Anat. Rec.*: **129**, 333.
HATERIUS, H. O.—(1930) *Ibid.*, **47**, 318.
IGLESIAS, R., MARDONES, E., BRUZZONE, S., AND LIPSCHUTZ, A.—(1953) *Arch. Anat. micr. Morph. exp.*, **42**, 3.
JONES, E. C. AND KROHN, P. L.—(1959) *Nature*, **183**, 1155.

LIPSCHUTZ, A.—(1931) *Endokrinologie*, **9**, 258.—(1937) *Gynéc. et Obstét.*, **36**, 407, 481.—(1938) *Ibid.*, **37**, 17.—(1941) *Arch. Path.*, **31**, 702.—(1950) ‘Steroid Hormones and Tumors’. Baltimore (Williams & Wilkins).—(1957) ‘Steroid Homeostasis, Hypophysis and Tumorigenesis’. Cambridge (Heffer).—(1958) *Acta Un. int. Cancr.* (in press).
*Idem*, KALLAS, H., AND R. PAEZ.—(1929) *Pflügers Arch. ges. Physiol.*, **221**, 695.
*Idem* AND OSNOWIKOFF, B.—(1932) *C. R. Soc. Biol., Paris*, **111**, 350.
*Idem*, ROJAS, G., CERISOLA, H. AND IGLESIAS, R.—(1958) *Acta Un. int. Cancr.* (in press).
*Idem* AND VOSS, H. E.—(1925) *Brit. J. exp. Biol.*, **3**, 35.
LOEB, L.—(1948) *Arch. Path.*, **46**, 401.
MARDONES, E., AND LIPSCHUTZ, A.—(1956) *Brit. J. Cancer*, **10**, 517.
MORATÓ, J.—(1941) *Endocrinology*, **29**, 6191.
MÜHLBOCK, O.—(1957) *Ciba Found. Coll. on Ageing*, **3**, 115.
NADEL, E. M.—(1949) *J. nat. Cancer Inst.*, **9**, 271.
NELSON, W. O.—(1930) *Anat. Rec.*, **45**, 272.
OLCOTT, C. T., AND PAPANICOLAOU, G. N.—(1943) *Cancer Res.*, **3**, 321.
OSNOWIKOFF, B.—(1934) *An. Med. interna*, **3**, 735.
PAPANICOLAOU, G. N., AND OLCOTT, C. T.—(1940) *Amer. J. Cancer*, **40**, 310.—(1942) *Arch. Path.*, **34**, 218.
PONSE, K., AND DOVAZ, R.—(1951) *Ann. Endocr., Paris*, **12**, 150.
RIESCO, A., AND SCHWARZ, J.—(1944) *Bol. Soc. Biol. Santiago de Chile* **1**, 69.
ROGERS, J. B.—(1951) *J. Geront.*, **6**, Suppl. 3, 142.—(1952) *Ibid.*, **7**, No. 3.
THUNG, P. J.—(1956) Experimental Research on Ageing, Symp., Basel. (Birkhäuser, Basel), p. 74.—(1958) ‘Ovaria van oude muizen’. Thesis, University, Amsterdam, 1958.
*Idem.*, BOOT, L. M., AND MÜHLBOCK, O.—(1956) *Acta Endocr., Copenhagen*, **23**, 8.
WARREN, S., AND GATES, O.—(1941) *Cancer Res.*, **1**, 65.
WOOLLEY, G. W.—(1950) *Recent. Progr. Hormone Res.*, **5**, 383.